# Structure of a pentameric virion-associated fiber with a potential role in Orsay virus entry to host cells

**DOI:** 10.1371/journal.ppat.1006231

**Published:** 2017-02-27

**Authors:** Yanlin Fan, Yusong R. Guo, Wang Yuan, Ying Zhou, Matthew V. Holt, Tao Wang, Borries Demeler, Nicolas L. Young, Weiwei Zhong, Yizhi J. Tao

**Affiliations:** 1 Department of BioSciences, Rice University, MS-140, Houston, Texas, United States of America; 2 Verna & Marrs McLean Department of Biochemistry & Molecular Biology, Baylor College of Medicine, One Baylor Plaza, Houston, TX, United States of America; 3 The University of Texas Health Science Center at San Antonio, Department of Biochemistry, MC 7760, 7703 Floyd Curl Drive, San Antonio, Texas, United States of America; 4 Department of Molecular and Cellular Biology, Baylor College of Medicine, One Baylor Plaza, Houston, TX, United States of America; Institut Pasteur, FRANCE

## Abstract

Despite the wide use of *Caenorhabditis elegans* as a model organism, the first virus naturally infecting this organism was not discovered until six years ago. The Orsay virus and its related nematode viruses have a positive-sense RNA genome, encoding three proteins: CP, RdRP, and a novel δ protein that shares no homology with any other proteins. δ can be expressed either as a free δ or a CP-δ fusion protein by ribosomal frameshift, but the structure and function of both δ and CP-δ remain unknown. Using a combination of electron microscopy, X-ray crystallography, computational and biophysical analyses, here we show that the Orsay δ protein forms a ~420-Å long, pentameric fiber with an N-terminal α-helical bundle, a β-stranded filament in the middle, and a C-terminal head domain. The pentameric nature of the δ fiber has been independently confirmed by both mass spectrometry and analytical ultracentrifugation. Recombinant Orsay capsid containing CP-δ shows protruding long fibers with globular heads at the distal end. Mutant viruses with disrupted CP-δ fibers were generated by organism-based reverse genetics. These viruses were found to be either non-viable or with poor infectivity according to phenotypic and qRT-PCR analyses. Furthermore, addition of purified δ proteins to worm culture greatly reduced Orsay infectivity in a sequence-specific manner. Based on the structure resemblance between the Orsay CP-δ fiber and the fibers from reovirus and adenovirus, we propose that CP-δ functions as a cell attachment protein to mediate Orsay entry into worm intestine cells.

## Author summary

Orsay, the only virus known to naturally infect the nematode *Caenorhabditis elegans*, expresses a capsid-δ fusion protein. We have demonstrated that the Orsay fusion protein is incorporated into Orsay capsid and forms a ~420-Å long fiber protruding from the capsid surface. Crystal structure of an N-terminal fragment of the δ sequence shows a pentameric coiled coil, suggesting that the Orsay fiber is incorporated into viral capsid at five-fold vertices. Our model predicts that δ consists of three domains: an N-terminal helical domain that is covalently linked to the capsid, a middle shaft that is mostly β-stranded, and a C-terminal head that likely hosts the cell receptor binding site for host attachment. Our findings have substantially advanced our understanding of the infection mechanism of this new virus. The Orsay δ protein represents the first pentameric viral fibers reported up-to-date with potential bioengineering applications.

## Introduction

For the past four decades, the nematode *Caenorhabditis elegans* (*C*. *elegans*) has been used as an important model organism for studying biological processes such as development, metabolism, aging, cell cycle and gene regulation [1]. However, it was not until the year 2011 that Orsay, the first virus that naturally infects *C*. *elegans* in the wild, was discovered [2]. Two other nematode viruses, Santeuil and Le Blanc, which both infect *C*. *briggsae*, have also been identified [2, 3]. Infections by these newly identified viruses cause abnormal intestinal morphologies without obvious effects on longevity or brood size [2, 4].

The Orsay virus has a bipartite, positive sense RNA genome of ~6.3 kb with three open reading frames (ORFs), including the putative RNA-dependent RNA polymerase (RdRP), the viral capsid protein (CP), and a nonstructural protein δ [2]. A plasmid-based reverse genetics system directly using the worm host has been developed for the Orsay virus [5]. Considering the simplicity of Orsay and the many advantages of *C*. *elegans* as a widely used model organism, Orsay-*C*. *elegans* serves as an excellent model for dissecting important mechanisms related to virus replication, virus-host interaction and host innate antiviral responses.

Phylogenetic analyses indicated that these nematode viruses are related to nodaviruses that infect primarily insects and fish [6]. Further molecular characterization revealed several fundamental differences compared to nodaviruses. For instance, translation of the Orsay CP utilizes an AUG-independent mechanism [7]. The nematode viruses also lack the subgenomic RNA3 found in nodaviruses that is used to express the nonstructural proteins B1/B2. The nonstructural B1 and B2 proteins encoded by both alpha- and betanodaviruses [8–10] function as either a host RNA interference (RNAi) suppressor or an anti-necrotic death factor to modulate cell death during virus infection [9–15]. In particular, alphanodavirus B2 folds into an α-helical structure that dimerizes in solution and binds dsRNA in a sequence-independent manner [16–18]. However, a recent study showed that the Orsay nonstructural protein δ does not possess any RNAi suppression activity [19].

Recent studies of the Orsay infection indicate that the RNAi pathway plays an important role in the *C*. *elegans* antiviral response. While the wildtype N2 strain only supports limited infection, inactivation of genes in the RNAi pathway such as *rde-1* [2] and *drh-1* [20] can sensitize N2 worms to Orsay infection. In *C*. *elegans*, RNAi following Orsay infection is not systemic, and there is no transgenerational inheritance of Orsay virus-induced silencing [21], different from the RNAi induced by exogenous dsRNA. In addition to RNAi, the ubiquitin-mediated defense also promotes Orsay resistance in N2 worms as revealed by a recent transcriptome study [22].

Recombinant Orsay CP is able to self-assemble into virus-like particles (VLP) in expression host. The crystal structure of an Orsay VLP has been determined, which displays a T = 3 icosahedral symmetry with 60 trimeric spikes [23]. Each Orsay CP can be divided into three linear regions, namely the N-terminal arm, the S domain forming the continuous capsid shell, and the P domain which forms trimeric surface protrusions. The Orsay CP is structurally distinct from alphanodaviruses (*e*.*g*. Flock House virus) [24–27], but has a structural fold closely resembling that of the betanodavirus [28].

The function of the Orsay δ protein remains a mystery. It was recently demonstrated that during infection δ could be expressed as a CP-δ fusion protein, which is likely generated by ribosomal frameshifting at the end of the CP ORF [7]. The RNA structure motif mediating the ribosomal frameshifting is conserved in all three nematode-infecting viruses [7]. The CP-δ fusion protein was detected in both infected worms and purified virion samples [7], but the expression of free δ has yet to be confirmed. Primary sequence alignment of the Orsay δ to those from Santeuil and Le Blanc gives 37% and 39% overall identity, respectively, suggesting an overall conserved structure and function.

To better define the functional role of the Orsay δ and CP-δ during infection, here we report the structure of these two proteins using both X-ray crystallography and electron microscopy (EM). Our results show that recombinant δ forms a fibrous molecule with a C-terminal globular domain. The N-terminal region of δ forms a pentameric α-helical bundle, but the rest of the protein is most likely β-stranded as suggested by sequence analysis and CD spectroscopy. The pentameric nature of the full-length δ fiber has been independently verified by both mass spectrometry and analytical ultracentrifugation. Coexpressing CP and CP-δ in insect cells produced Orsay VLPs with an enhanced amount of CP-δ compared to native virions. These VLPs were found to have multiple long fibers protruding from the capsid surface when observed under EM, indicating that the δ sequence adopts the same fibrous structure in both CP-δ and free δ. Considering its five-fold symmetry, the CP-δ fibers are expected to occupy five-fold vertices in the capsid. Furthermore, reverse genetics confirmed that the structural integrity of CP-δ is essential for Orsay infection, and competition assays showed that purified δ proteins, when added *in trans*, effectively inhibit Orsay infection. By analogy with other viral capsid-associated fiber proteins, the Orsay CP-δ likely functions in cell entry as a cell receptor binding protein.

## Results

### Recombinant δ forms a 420-Å long fiber with a C-terminal head domain

**Fig 1:**
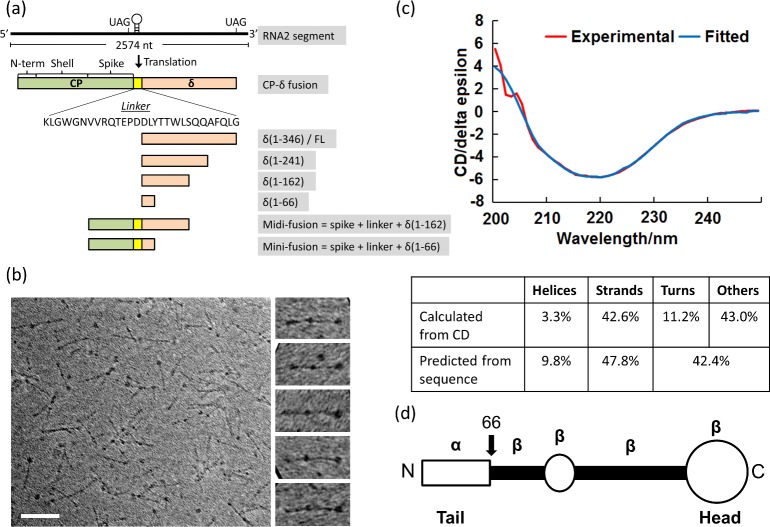
Orsay δ protein. (a) The coding scheme of Orsay δ and CP-δ fusion protein. CP-δ, which is produced by ribosomal frameshifting, is comprised of the full-length CP, a 29-aa linker, and the full-length δ. The Orsay CP can be divided into three parts, the N-terminal peptide, shell, and the spike domain. The five δ and CP-δ truncation mutants are shown below. (b) TEM images of δ by negative staining. On the right are five enlarged δ aligned in the horizontal direction. Scale bar, 500 Å. (c) CD spectra of δ. The experimental data and the fitting curves are shown in red and blue, respectively. The table below compares the secondary structure contents calculated from CD and sequence-based prediction. (d) δ domain map. There is a slightly enlarged tail domain at the N-terminus (aa1-66), a small globular domain in the middle, and a large globular head at the C-terminus.

**Fig 2:**
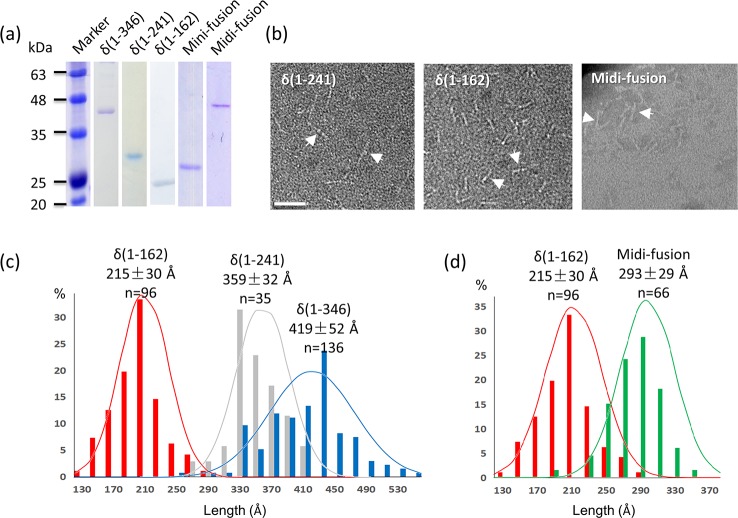
Truncation mutants of δ and CP-δ. (a) SDS-PAGE showing five purified proteins. (b) Negative-staining EM images. δ(1–241), δ(1–162) and the midi-fusion all formed fiber-shaped molecules. Some of these fibers are highlighted by arrows. Scale bar, 500 Å. (c) Length measurements for the two δ truncation mutants in comparison with the full-length δ. (d) Length measurements for the midi-fusion protein in comparison with δ(1–162).

To characterize the structure and functions of Orsay δ/CP-δ, the δ ORF was cloned into a pET vector for overexpression in *E*. *coli*. A 6×His SUMO tag was added at the N-terminus of the full-length δ to facilitate protein purification (Fig 1A). Recombinant δ was expressed as a soluble protein and ~60% could be recovered in the cytoplasmic fraction. When subjected to a Superdex-200 gel filtration column, δ produced a sharp peak at ~58 ml, corresponding to an apparent molecular size of ~670 kDa, which is substantially higher than the calculated molecular mass of a monomer (*i*.*e*. 38.4 kDa) (S1 Fig). Negative-staining transmission electron microscopy (TEM) was then performed to examine the molecular organization of δ. Surprisingly, TEM images showed that δ forms fibrous molecules of extended length (Fig 1B), thus explaining its unusual elution profile from the size exclusion column. Based on the measurement of 136 molecules, the length of the δ fibers was determined to be ~419±52-Å (Figs 1B and 2C). Close examination of individual δ molecules reveals several fine structural details: (1) at one end there is a globular head domain with a diameter of ~50-Å; (2) a second globule, which is slightly smaller, is found at roughly two-fifths of its length; (3) the thickness of the fiber is only ~20-Å in most parts; and (4) the other end of the fiber opposite to the large head domain often appears to be slightly enlarged in diameter (Fig 1B). There was no particular bending point observed along the fiber, but the fibrous region near the globular head often exhibited more pronounced curvatures. Other than the ~420-Å δ fibers, longer or thicker filaments were not observed in EM, suggesting that further oligomerization of the δ fiber did not occur.

Using bioinformatics tools PSIPRED [29] and Prof [30], the secondary structure of δ was predicted based on its amino acid sequence (S2 Fig). The N-terminal 65 residues were predicted to form an α-helical structure, whereas the rest of δ was predicted to be primarily β-stranded. The online server Motif Scan [31] also detected a valine-rich region in the middle of the Orsay δ sequence that is known to form regular arrays of β-strands in a number of exo/cytoskeleton-related proteins including putative insect cuticle proteins and a putative adhesin in *Parabacteroides distasonis* (PDB ID: 3LJY). Circular dichroism (CD) was measured to experimentally determine the secondary structure content of δ (Fig 1C). The CD spectra indeed showed a single trough near 220nm, which is highly characteristic for β-stranded structures, consistent with the results from secondary structure prediction. A quantitative analysis of the CD spectra was done using the program BeStSel [32], which estimated that δ is composed of 3.3% α-helices and 42.6% β-strands (Fig 1C).

Considering its uniform length and non-repetitive structural features, each fiber molecule in the TEM images is likely a linear oligomer of δ that is arranged in either a parallel or an anti-parallel manner (Fig 1B). To test this hypothesis, a number of δ truncates were designed with progressively larger amounts of N- and C-terminal sequences removed. While the N-terminally truncated mutants, including δ(167–346), δ(195–346), δ(220–346), δ(241–346) and δ(256–346), were poorly soluble, all C-terminally truncated mutants behaved similarly to the full-length δ during purification and were eluted as a major peak from the gel filtration column (Fig 1A and S1 Fig). TEM further confirmed that both δ(1–162) and δ(1–241) formed fibrous molecules, however, their lengths were shorter than the full-length protein, with δ(1–162) and δ(1–241) fibers measured to be 215±30-Å (n = 96) and 359±32-Å (n = 35) long, respectively (Fig 2B and 2C). Considering that the full-length δ protein is ~420-Å long, the length of these two mutants is roughly proportional to the size of their respective sequence. The large globular head domain was absent from both δ(1–162) and δ(1–241), indicating that the head domain is formed by the C-terminal sequence. Overall, our results suggest that the δ protein fiber is a parallel oligomer with a C-terminal head domain, because these two δ protein truncates formed fibrous molecules resembling the left end of the δ protein fiber (Fig 1D).

### δ sequence in CP-δ also forms a fibrous structure

It has been shown that the Orsay δ ORF could be translated as a CP-δ fusion protein by ribosomal frameshifting and the CP-δ was observed in infected cells as well as in purified virion samples [7]. In purified virions, the amount of CP-δ only counts for ~5% of the total CP [7]. While fusion proteins are frequently encoded by RNA viruses to regulate non-structural protein expression, fusion proteins as structural components are rarely observed in RNA viruses. To our knowledge, the only known exception is totiviruses (*e*.*g*. yeast LA virus), a group of dsRNA viruses with non-segmented genomes, which express the viral RNA polymerases as *gag-Pol* fusion proteins that are incorporated into viral particles at low copy numbers [33].

To determine whether the δ sequence in CP-δ assumes a similar or different structure compared to the free δ, we subcloned the CP-δ sequence for recombinant protein expression. By inserting a single nucleotide “A” in front of the last nucleotide before the stop codon of the CP gene, we were able to position the δ ORF in the same coding frame as the preceding CP ORF (Fig 1A). However, the Orsay CP-δ was found to be insoluble when expressed in either *E*. *coli* or insect cells. Because Orsay CP has a strong tendency to assemble into VLPs [23], the most likely explanation for the solubility problem is that the different oligomerization behaviors of the CP component (*i*.*e*. dimer, trimer, pentamer, and hexamer) and the δ component (*i*.*e*. pentamer–see below) of the fusion protein resulted in an infinite molecular network and thus the formation of large aggregates.

In an effort to resolve the solubility issue, we constructed a mini-fusion protein (Fig 1A). The mini-fusion protein, also called CP-δ(215–485), is comprised of the CP spike domain, a 29-aa linker, and the first 66 residues of the δ protein that were predicted to form α-helices (Figs 1A and S2). The CP spike domain forms trimeric surface protrusions [23], but is not able to oligomerize any further in the absence of the rest of the CP polypeptide. When expressed in *E*. *coli*, the mini-fusion protein was soluble with an apparent MW of ~150 kDa based on gel filtration chromatogram, consistent with the theoretical calculation for a pentameric assembly (Figs 2A and S3). Considering that the mini-fusion protein is too short for EM observation, we next expressed and purified a midi-fusion protein, which contains the CP spike domain, the 29-aa linker, and the first 162 residues of δ (Figs 1A and 2A).

When the purified midi-fusion protein was subjected to negative-staining EM, fibrous molecules were again observed with a morphology similar to that of δ(1–162) (Fig 2B). The length of the midi-fusion protein is about 293±29-Å (n = 66), which is slightly longer than the δ(1–162) fiber (*i*.*e*. 215±30-Å) (Fig 2D). The presence of the CP spike domain, which is ~35-Å in height according to the structure of the VLP, likely counts for the length discrepancy between the midi-fusion protein and δ(1–162). This finding led us to conclude that δ in the CP-δ fusion protein also adopts a fibrous structure similar to the free δ. It remains to be determined whether the 29-aa linker sequence adopts a particular conformation with a specific function or simply acts as a flexible linker.

### δ(1–66) forms a pentameric helical bundle

**Fig 3:**
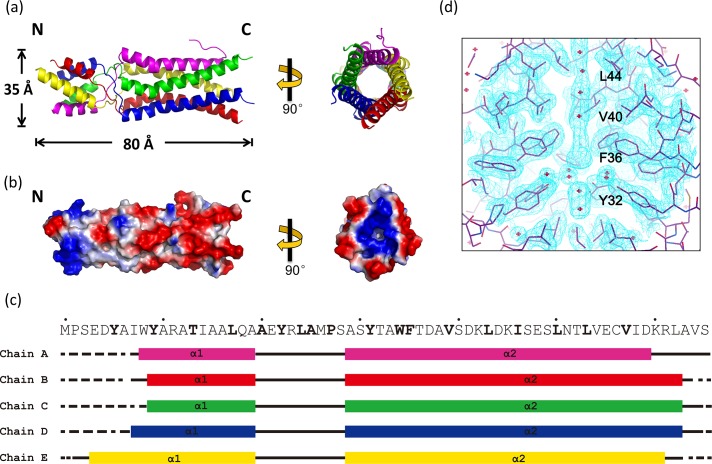
Crystal structure of δ(1–66). (a) Ribbon diagram. The five subunits are shown in different colors. (b) Surface representation colored by electrostatic potential. Both a side-view (left) and a top view (right) are provided in (a) and (b). (c) Secondary structure for the five chains in a pentamer. Bold letters highlight the residues with their side chains pointing to the interior of the helical bundle. α-helices are shown by boxes, non-structured loops are represented by black lines, and disordered regions are shown by dotted lines. (d) An electron density map showing water-like features inside the helical bundle.

Considering the extended shape and flexible nature of the δ fiber, crystallization of the full-length δ protein would be highly challenging if not impossible. Therefore, several deletion mutants of δ, including δ(1–66), δ(1–138), δ(1–162) and δ(1–241), were purified and subjected to crystallization. Among these four constructs, only δ(1–66), which contains the first 66 residues of δ, produced crystals diffracted to better than 3-Å resolution. The structure of δ(1–66) was determined by single-wavelength anomalous dispersion (SAD) using SeMet-substituted crystals (Table 1). By subsequent molecular replacement and refinement against a native dataset, we were able to establish the final structure to 2.2-Å resolution (Table 1, Fig 3).

**Table 1:** Crystallographic statistics.

	Native δ(1–66)	SeMet-substituted δ(1–66)
Data collection		
*Wavelength (Å)*	0.97853	0.97857
Space group	C2	P2_1_2_1_2_1_
Resolution (Å)*	50.00–2.22 (2.26–2.22)	50.00–3.30 (3.36–3.30)
Cell dimensions		
a, b, c (Å)	175.31, 32.12, 65.62	33.05, 91.22, 118.82
α, β, γ (°)	90, 100.02, 90	90, 90, 90
Unique reflections	17,899	5,877
Redundancy*	4.4 (3.7)	13.9 (14.5)
Mean I/σ (I)*	12.7 (2.2)	31.7 (5.1)
R_merge_ (%)*	11.8 (43.5)	9.8 (44.4)
Completeness (%)*	97.8 (85.2)	100.0 (100.0)
Phase determination		
Number of HA sites	5	
Figure of merit	0.242	
Refinement		
R_work_/R_free_	20.64/23.14	
R.m.s. deviations		
Bond lengths (Å)	0.008	
Bond angles (°)	0.846	
Ramachandran statistics ^‡^		
Preferred regions	278 (97.89%)	
Allowed regions	6 (2.11%)	
Outliers	0 (0.00%)	

*Numbers in parenthesis are for the highest resolution shell.

^‡^Calculated in Coot.

δ(1–66) assembles into a pentamer with the five subunits forming an α-helical bundle (Fig 3A). This five-helical bundle is ~80-Å long and ~35-Å wide (Fig 3A and 3B). Each δ(1–66) molecule folds into two α-helices that are connected by a 9-aa linker (*i*.*e*. residues 21 to 29) (Fig 3C). The longer α-helix, consisting of residues 30 to 63, has a kink at around residue 40 (Fig 3A). The helix after the kink contains three regular heptad repeats (*i*.*e*. ^40^**V**SDK**L**DK**I**SES**L**NT**L**VEC**V**ID^60^, in which hydrophobic residues are highlighted in bold). Heptad repeats are frequently observed in coiled coil structures (*i*.*e*. dimeric, trimeric, tetrameric and pentameric) and they contain amino acid sequences arranged in the periodicity of (*a b c d e f g*), with positions *a* and *d* predominantly occupied by hydrophobic residues. Hydrophobic side chains at the positions *a* and *d* make up a continuous hydrophobic surface on the α-helix so that multiple α-helices can wrap around each other to form a stable helical bundle [34, 35]. Surface representation of the δ(1–66) pentamer shows a ~3 to 5-Å wide channel running through the entire molecule. A total of 18 residues are found to have their side chains pointing towards the interior of the channel, including Y6, Y10, T14, L18, A21, Y23, L25, A26, P28, Y32, W35, F36, V40, L44, I47, L51, L54, and V58 (Fig 3C and 3D). Therefore, the core of the entire helical bundle is mostly hydrophobic, except for a single location at T14. In the electron density map, blobs of densities that are modeled as water molecules occupy the central channel (Fig 3D). The large hydrophobic cavities at the center of the helical bundle may help to accommodate these water molecules [36].

It has been reported that the cartilage oligomeric matrix protein contains a five-stranded coiled-coil domain with a continuous axial pore with binding capacities for hydrophobic compounds, including prominent cell signaling molecules [37]. It remains to be found whether the Orsay N-terminal helical bundle has any specific ligand binding activity like the cartilage oligomeric matrix protein. The symmetry and the shape of the δ(1–66) structure also bear some resemblance to a class of pentameric viroporins, such as the small hydrophobic SH protein encoded by human respiratory syncytial virus [38] and the E protein found in SARS-CoV [39]. Ion channel activities, however, do not seem to apply to δ, as δ expression in *E*. *coli* is not associated with cytotoxicity and overexpressed δ is predominantly cytoplasmic instead of membrane-bound, which are markedly different from the reported behaviors of known viroporins [40].

### Full-length δ forms unique pentamers among virion-associated fibers

**Fig 4:**
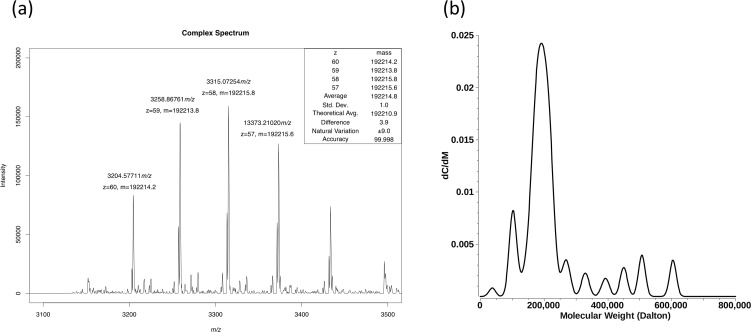
Full-length δ forms exclusive pentamers. (a) Mass spectrometry confirms the predominance of the homo-pentameric complex. The experimental mass of the intact complex is 192214.8 ±1 Da and is almost exactly the mass expected for a homo-pentamer. There is no indication in the mass spectrum of the existence of an alternative stoichiometry, including monomer. Note that due to the natural variance in isotopic composition the average mass of a protein complex of this size is inherently uncertain. The sum of the uncertainties of the relative atomic masses for this composition results in an estimate of the natural variation of approximately ±9Da, indicating that the observed difference of approximately 4 Da is well within the range expected. (b) Molar mass analysis by analytical ultracentrifugation. The integrated molar mass of the major peak is consistent with a pentameric species. Higher molecular weight species are also visible, but are present at lower concentration. Molecular species > 800,000 Da are not shown for clarity. Minor peaks may explained by modeling inaccuracies and/or contaminating proteins.

To confirm that the full-length δ fiber is a pentamer as indicated by the δ(1–66) crystal structure, mass spectrometry was used to analyze the molecular weight of the molecule under non-denaturing conditions. Under even relatively energetic conditions a mass consistent with a homo-pentamer (192214.8 ±1 Da) was the only major mass observed (Fig 4A). This compares favorably to the average mass according to the sum of the relative atomic masses of 192210 Da and to the extremes of isotopic composition of 192187.4 and 192226.5 Da, as reported by IUPAC (2013 revision). It is important to note that neither the monomeric species, nor any other multimeric species, was observed even under the relatively energetic conditions shown (and all conditions tried). This indicates that the homo-pentamer is exceptionally stable.

Under sufficiently energetic conditions, the gas phase pentameric complex will dissociate upon collision with buffer gas to produce exclusively monomers. No intermediates were observed with minimal covalent bond cleavage (S4A Fig). This resulted in a protein of observed mass 38441.1 Da which again compares well with the theoretical average mass of a monomer according to the sum of the relative atomic masses of 38442.2 Da and to the extremes of isotopic composition of 38437.5 to 38445.3 Da, as reported by IUPAC (2013 revision). Thus, the direct observation of pentamers and their dissociation to exclusive monomers upon sufficient activation energy confirm that the original complex is a homo-pentamer of high stability. The absence of alternate stoichiometries, either prior to dissociation or as a product or intermediate of the dissociation process, further supports the pentamer as the likely near exclusive stoichiometry.

Resolution of the isotopic distribution of the monomer mass spectrum confirms the assigned charge state and gives another estimate of mass. This mass of 38440.12 Da is based on the most abundant isotope and fundamentally differs slightly from the average (S4B Fig). Note that although greater accuracy is achieved here, the accuracy of all of the mass measurements presented are far more accurate than the inherent variance in mass due to the natural variation in isotopic composition found in different environments.

Sedimentation velocity experiments (SV) were also used to study the Orsay virus full-length δ protein in solution. SV experiments characterize the solution behavior of macromolecules and observe the sedimentation and diffusion behavior of all species in a mixture, and report their partial concentrations, buoyant molecular weights, and anisotropies. Sedimentation coefficient distributions from the δ protein demonstrated the presence of a major species sedimenting with a fairly broad peak centered at 5.4 s with a frictional ratio of 2.1, which indicates a high degree of anisotropy (S5 Fig). This is consistent with a fibril-like conformation of the protein. A molar mass transformation of this peak resulted in a weight-average molar mass of 191.0 kDa, in excellent agreement with the calculated molecular weight of 192.2 kDa for the pentameric form of this protein (Fig 4B, Table 2).

**Table 2:** Integration results from the PCSA-DS.

Molar mass (kDa) (measured)	Molar mass (kDa) (theoretical)	*s* (x 10^−13^ sec)	*f/f*_*0*_	Relative amount
191.2 (121.2, 261.2)	192.2	5.4 (3.7, 7.1)	2.1 (2.0, 2.3)	68.2%

Values in parenthesis are 95% confidence intervals from the peak integration. This transformation to absolute molar mass is based on an estimated partial specific volume of 0.740 ml/g.

### Recombinant Orsay capsid containing CP-δ shows protruding long fibers

**Fig 5:**
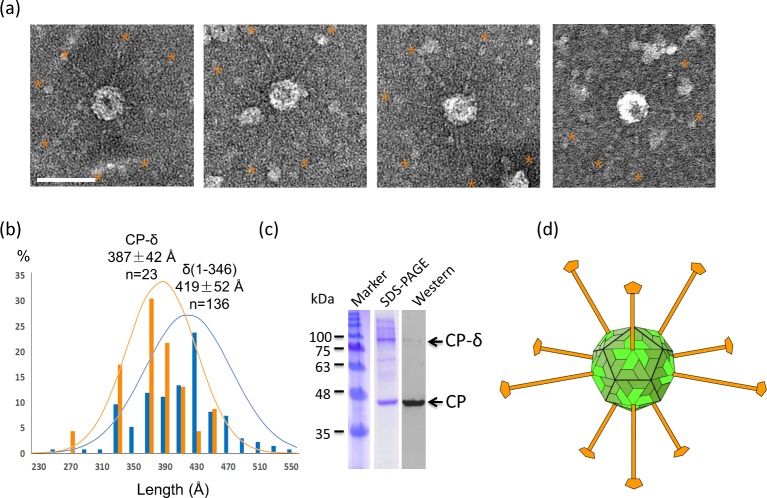
CP-δ forms particle associated fibers. (a) EM images of Orsay VLPs containing CP-δ. Protruding fibers are highlighted by orange stars. Scale bar, 500 Å. (b) Length measurements of particle-associated fibers in comparison with the full-length δ fibers. (c) SDS-PAGE of the Orsay VLP. Western blot was performed using an anti-His antibody. (d) An Orsay capsid model with CP-δ fibers situated at the five-folds. On five-fold vertices, the CP portion and the δ portion of the CP-δ are shown in light green and orange respectively. The rest of the CP molecules in the capsid are shown in dark green. The Orsay, ~350-Å in diameter, and the CP-δ fiber, ~400-Å in length, are approximately drawn to scale.

To analyze the structure of CP-δ in the context of a viral capsid, we co-expressed CP and CP-δ in insect cells by co-infection with two baculoviruses each expressing a different protein. The use of two baculoviruses would allow the control of the relative amount of CP and CP-δ to optimize particle assembly. Recombinant VLPs were purified by Ni-NTA affinity as both CP and CP-δ contained a C-terminal His-tag. Under negative-staining EM, we observed many spherically shaped particles associated with long fibers (Fig 5A). The diameter of these particles is around 350-Å, closely matching that of the Orsay viron or VLP [2, 23]. The length of particle-associated fibers, when measured from the surface of the capsid, is 387±42-Å (n = 23), which is similar to the length of free δ measured at 419±52-Å (Fig 5B). Some of the fibers even show a head domain at their distal end, consistent with our assumption that the N-terminal coiled coil of the δ fiber is directly attached to the CP surface spike. Considering the 5-fold symmetry of the δ protein, in principle there can be up to 12 copies of the CP-δ fibers in each capsid, with one occupying each icosahedral vertex (Fig 5D). Coomassie-stained SDS-PAGE gel of our capsid sample showed the mass ratio of CP-δ to CP is around 1:2 (Fig 5C), which corresponds to roughly 1:4 in molar ratio, suggesting that on average there should be ~7 pentameric fibers in each particle. The most fibers we observed in a single VLP were seven. It is possible that some fibers were not visible due to staining artifacts, or that not all CP-δ was properly incorporated into capsids.

### Structural integrity of CP-δ is critical for Orsay infectivity

**Fig 6:**
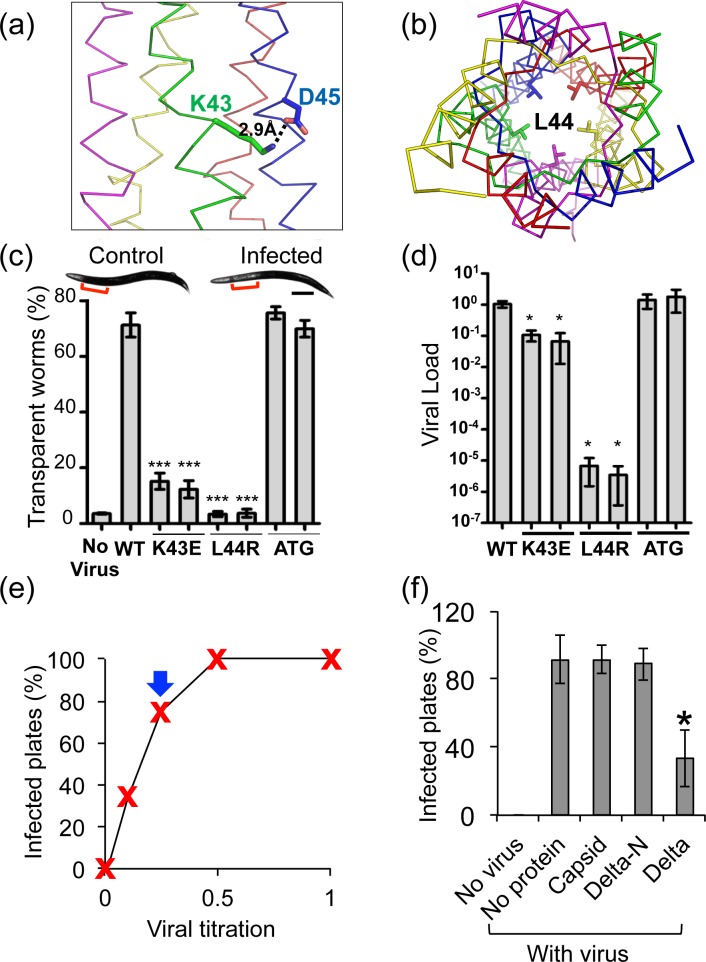
Site-directed mutagenesis and infectivity assays. (a) K43 forming a salt bridge with D45. Only a portion of the structure is shown. (b) L44 facing the hydrophobic interior of the helical bundle. (c) Transparency test for wild-type (WT) and mutant Orsay viruses. As displayed in the inset, Orsay-infected worms, but not the control, show a transparent intestine particularly at the anterior region as indicated by red brackets. Scale bar, 100μm. (d) Viral load of wild-type and mutant viruses measured by qRT-PCR. In (c-d), two independent transgenic lines for each mutant virus were tested. ATG represents the δ-null mutant. Three independent trials (biological replicates) were conducted. Error bar, standard error from three biological replicates. *, p<0.05; ***, p<0.001 compared with WT using Student’s t-test. (e) A typical viral titer determination result. The arrow indicates the viral concentration used for the protein-competition assay. (f) Adding full-length δ in the culture medium reduces viral infectivity. Delta-N represents the δ(1–101) deletion mutant. Bars and error bars show mean and standard deviation from three independent trials. 12 plates were tested in each trial for each protein/condition. *, p<0.05, Student’s t-test, paired samples.

Site-directed mutagenesis and reverse genetics were performed to confirm that the structural integrity of CP-δ is important for Orsay infectivity. Two residues K43 and L44 were targeted for mutation. The crystal structure of δ(1–66) shows that K43 and D45 form an intermolecular salt bridge on the surface of the α-helical bundle (Fig 6A). L44 is located at the hydrophobic core of the pentameric coiled coil (Fig 6B). Both mutations K43E and L44R were expected to disrupt the structure of δ/CP-δ. Indeed, our results showed that δ(1–66) constructs bearing either the K43E or the L44R mutation could no longer form regular pentamers, considering the substantial shifts in their peak positions in gel filtration profiles (S6 Fig).

Using transgenic *C*. *elegans* carrying virus cDNAs as previously described [5], two recombinant viruses, one with the K43E mutation and the other with the L44R mutation, were generated. Recombinant viruses collected from transgenic worm lysate were applied on naïve worms sensitive to Orsay infection. These worms were then evaluated for viral infectivity by two measurements: infection symptoms and viral load. Orsay-infected worms often display a transparent intestine phenotype (Fig 6C). The percentage of worms with such symptom was drastically reduced with the two mutant viruses (Fig 6C). The L44R mutant virus appeared more defective than K43E, as worms infected with the L44R virus showed no difference than uninfected worms, while a small fraction of worms infected with the K43E virus showed the infection symptom (Fig 6C). These observations were confirmed in multiple independent lines of transgenic worms (Figs 6C and S7, demonstrating that the differences were indeed caused by viral genotypes. In addition, the viral load in these worms was determined using qRT-PCR, and consistent results were obtained (Fig 6D). Both mutations significantly reduced the viral load, with more severe defects with the L44R mutant virus.

As the K43E and the L44R mutations could potentially affect both CP-δ and free δ, another recombinant Oray virus was generated to distinguish which protein led to the observed defects. In this recombinant virus (*i*.*e*. δ-null), the start codon of free δ was mutated from ATG to CTG so that no free δ was produced. A high percentage of worms infected with this mutant virus showed the transparent intestine symptoms; the viral load was also similar to that of wild-type virus (Fig 6C and 6D), suggesting that free δ was not required for infection based on our assay. Therefore, the K43E and the L44R mutant viruses lost their infectivity likely because of defective CP-δ.

### The addition of recombinant δ *in trans* inhibits Orsay infection

The lack of infectivity from δ mutants suggested that CP-δ is required for infection. We asked whether CP-δ functions in viral entry or at a later stage. We reasoned that if δ functions in viral entry steps such as receptor-binding at the cell surface, then adding purified δ in the culture medium would compete against the virus CP-δ for such binding sites, and would thus reduce the efficiency of Orsay infection. On the other hand, if CP-δ functions in steps post viral entry, such as intracellular viral replication, then adding proteins in the culture medium would have little impact on viral infectivity.

To conduct the protein-competition assay, we first determined the viral titer and chose the lowest viral concentration with over 70% infectivity (Fig 6E). At this viral concentration, adding 2μg/ml full-length δ to the culture medium significantly reduced the infectivity from 92% to 33% (Fig 6F), demonstrating that δ functions at the viral entry step. In contrast, adding purified capsid or the N-terminal fragment δ(1–101) did not show this effect (Fig 6F), consistent with our structural model that the δ C-terminal globular head functions in cell attachment.

## Discussion

Results from our study indicated that the CP-δ fusion protein plays a specific function in host cell entry during Orsay infection based on the following evidence: (1) Orsay δ forms pentameric fibers; (2) CP-δ is incorporated into viral capsid as a minor structural protein; (3) the δ portion of CP-δ forms a long projecting fibers with a globular head domain at the distal end; (4) disrupting the structural integrity of CP-δ results in non-viable virus mutants; and (5) the addition of recombinant δ to worm medium reduced Orsay infectivity. The use of recombinant VLPs enabled us to directly visualize the CP-δ fibers due to the enhanced amount of CP-δ in the VLP sample. In contrast, the native virion sample contains only ~5% CP-δ [7], which corresponds to only one to two CP-δ fibers in average in each particle. It would be difficult to identify these long fibers by EM unless they lie flat on sample grids and interact evenly with heavy atom stains, thus explaining the difficulties we had trying to visualize such fibers using native virion samples.

The Orsay δ/CP-δ fiber consists of several domains (Fig 1D). The first ~60 residues at the N-terminus of δ form an α-helical bundle and play an important role in stabilizing the δ/CP-δ pentameric fiber. The rest of the sequence is largely β-stranded and likely forms β-barrels or β-spirals connected by non-structured loops. While β-barrels and β-spirals are frequently observed in viral structural proteins, pentameric β-fibers have not been previously reported [41, 42]. The diameter of the β-fiber in the Orsay δ is only ~25-Å, smaller than that of the helical bundle at the tail end as shown by EM images. There is a large globular head at the C-terminal end of the δ/CP-δ fiber. Another globular domain, which is smaller in size, is found at the two-fifths position from the N-terminal end. The observation of a globular head at the distal end of the capsid-associated CP-δ fibers is consistent with our domain assignment.

Although we have not experimentally verified that the CP-δ fibers are also pentamers, the consideration of stereochemical constraints in the context of an Orsay capsid suggests that pentameric fibers are energetically favored. The crystal structure of the Orsay capsid shows that the C-terminus of the Orsay CP is tucked underneath of a tightly bound trimeric spike [23] (S8 Fig). Therefore, for a trimeric fiber to form, the polypeptide sequence would have to go around the timeric spike from outside, spanning a distance of at least 60-Å in order to reach the 3-fold axis. By comparison, the C-terminus of the CP points toward a depression around the 5-fold symmetry axis, with only a 25-Å traveling distance to the 5-fold, thus facilitating the formation of pentameric fibers. Our results from mass spectrometry also indicate that the pentameric δ fiber is very stable and does not dissociate unless under high energetic conditions, suggesting that it is unlikely for the δ sequence to adopt an alternative trimeric configuration in the form of the CP-δ fusion protein.

While δ showed no sequence homology to any known proteins, the morphology of the CP-δ protein, as well as its localization in the capsid and its secondary structure content, is reminiscent of the fibers found in both reovirus and adenovirus [43, 44]. Like CP-δ, reovirus σ1 fiber protein is organized into three modules: a coiled coil tail domain at the N-terminus, a β-filament body domain in the middle, and a C-terminal β-stranded head domain. The adenovirus fiber does not have a coiled coil region, but also has a head-and-tail morphology with a long shaft made of ~20 β-spiral repeats and a C-terminal head comprised of an eight-stranded β-barrel [45]. Both adenovirus and reovirus fibers are situated at five-fold symmetry axes with their N-terminal sequence interacting with the viral capsid and their C-terminal head at the distal end [46, 47], same as the Orsay CP-δ. The Orsay CP-δ forms pentameric fibers, however, while the adenovirus and reovirus fibers are both trimeric. In adenovirus and reovirus, the cell receptor binding sites are mapped to the globular head domain of their fibers, except that in some reoviruses a sialic acid binding site is found in the middle body domain of σ1 [48–51]. The overall lengths of the adenovirus (*i*.*e*. Ad2 and Ad5) and reovirus fibers are ~325 and 385-Å, respectively [45], slightly shorter than the Orsay CP-δ fiber.

Results from the competition experiments using free δ (Fig 6E and 6F) and the close analogy between the CP-δ fiber and the fibers from reovirus and adenovirus suggest that the Orsay CP-δ likely functions as a cell receptor binding protein. The globular head of CP-δ likely hosts the cell receptor binding site as it does in reovirus and adenovirus. The binding of CP-δ to the host receptor should allow virus attachment to the host intestinal cells for the subsequent cell entry. The cell receptor molecule for Orsay has yet to be determined, but we expect that viral particles containing only CP but no CP-δ fibers would be non-infectious due to blocked cell entry. It is unclear whether having only 1 to 2 copies of the CP-δ fiber instead of a full complement of 12 would negatively impact Orsay’s infectivity, but dsDNA bacteriophages such as ϕ29 are highly infectious with only one tail structure in each viral particle [52, 53]. For the bacteriophage T4, it was demonstrated that three fibers per virion are sufficient for infectivity, and reducing the lipopolysaccharide receptor concentration on cell surface has the same effect as tail fiber limitation on phage infectivity [54]. Therefore, it is possible that not all 12 copies of the CP-δ fiber are needed for Orsay, especially if abundant receptor molecules exist on the *C*. *elegans* intestinal cell surface.

Although our infectivity assays did not detect any obvious functional defects for the δ-null mutant, we cannot rule out the possibility that free δ may still play important roles during the virus life cycle that are distinct from the cell entry function mediated by CP-δ. Both of our infectivity assays in Fig 6, one based on the body transparency of infected animals and the other measuring viral RNA in worm lysates, relied on one infection cycle and therefore mainly detected mutant defects in viral entry. Mutant defects downstream from viral RNA replication cannot be effectively measured using these assays. It remains possible that the free δ protein may interact with the host machinery to promote virus assembly and/or mediate the release of viral particles from the apical side of the worm intestine cells. Furthermore, many non-enveloped animal viruses are known to encode a lytic peptide or protein, but such function has not yet been reported for Orsay. Free δ may function as a lytic protein. Future cytological and biochemical analyses should help to identify interesting leads in this direction.

By defining the structure and function of the Orsay CP-δ fibers, findings from our present study represent a major advance in our understanding of Orsay cell entry. Additionally, we expect our results to serve as a useful guide for future work related to Orsay host receptor identification as well as detailed characterization of the molecular interaction between Orsay and its host receptor.

## Materials and methods

### Molecular cloning

The coding sequence of the full-length Orsay δ (GenBank accession no. HM030971.2) was inserted into a modified pETDuet-1 vector that would add a 6xHis-SUMO tag to the recombinant protein at the N-terminus. Removal of the fusion tag using the SUMO protease Ulp should leave a dipeptide HM at the N-terminal end of the recombinant protein. C-terminal truncation mutants δ(1–66), δ(1–101), δ(1–162), and δ(1–241) were made by introducing a termination codon at desired sites by PCR using a pair of complementary primers.

To make CP-δ fusion protein constructs, a single nucleotide “A” was inserted in front of the last nucleotide before the stop codon of the CP ORF to shift the δ ORF to the same coding frame. The modified sequence would express CP-δ, the same as expected from ribosomal frameshifting. For the mini-fusion protein, the DNA sequence coding for residues 215–485 of CP-δ, which contains the protrusion domain of the CP, the 29-aa linker, and an N-terminal fragment of δ(1–66), was cloned into the modified 6xHis-SUMO pETDuet-1 vector as mentioned above, For the midi-fusion protein, the DNA sequence coding for residues 215–581 of CP-δ, which contains the protrusion domain of the CP, the 29-aa linker, and an N-terminal fragment of δ(1–162) together with a C-terminal 6xHis tag, was cloned into pFastBac1 (Thermo Fisher Scientific) and the recombinant baculovirus was subsequently generated following the Bac-to-Bac Expression System manual.

To produce recombinant Orsay capsid containing CP-δ, the DNA sequences coding for Orsay virus CP-δ fusion protein (C-terminally 6xHis-tagged) and CP (N- and C-terminally 6xHis- tagged) were each cloned into pFastBac1. Two baculoviruses were generated as described above, one expressing CP and the other expressing CP-δ.

### Protein expression and purification

For protein expression in *E*. *coli*, cells at the phase of exponential growth were induced using 1 mM Isopropyl β-D-1-thiogalactopyranoside (IPTG) when OD_600nm_ reached 0.6–0.8. After overnight shaking at 15°C, cells were harvested by centrifugation at 2,000xg for 20 min and sonicated in lysis buffer containing 50 mM Tris pH8.0, 300 mM NaCl, 10% glycerol (v/v), 5 mM 2-Mercaptoethanol (2-ME), 1 mM NaN_3_ and 1 mM phenylmethylsulfonyl fluoride (PMSF). 6xHis-SUMO-tagged proteins were first purified by affinity chromatography using the Ni-NTA resin (Thermo Fisher Scientific). After Ni-NTA affinity, the eluates were collected and incubated with a SUMO protease (Ulp) at a mass ratio of 1: 10 [Ulp: His_6_—SUMO - δ(1–66)] overnight at 4°C for affinity tag removal. Afterward, the mixture was brought to 25 mM imidazole and re-applied to Ni-NTA resin and the flow-through containing the δ(1–66) from the second Ni-NTA was collected. The sample was next purified by size exclusion using a Superdex 200 gel filtration column that with an elution buffer containing 50 mM Tris-HCl (pH 7.5), 250 mM NaCl, 350 μl 2-ME, and 1 mM NaN_3_. Peak fractions containing δ(1–66) were loaded onto a 2-ml HisTrap HP column (GE Healthcare Life Sciences) for a final cleanup. The flow-through containing purified proteins was concentrated to 5 mg/ml and stored at 4°C. SeMet-substituted δ(1–66) was expressed in M9 minimal medium supplemented with SeMet [55]. Expression was induced with 1 mM IPTG for 24 h at 15°C.

For the midi-fusion protein, ~2X10^8^ (or 200 ml) *Spodoptera frugiperda* 21 (Sf21) insect cells grown in supplemented Grace’s insect medium (Life Technologies) were infected with baculovirus and harvested 60 h post-infection. The cell pellets were washed with cold phosphate-buffered saline (PBS) and sonicated in a cold lysis buffer containing 50 mM Tris-HCl (pH 8.0), 300 mM NaCl, 1 mM NaN_3_, 1 mM PMSF, 10% (v/v) glycerol, 0.5% (v/v) Triton X-100, 10 μg/ml DNase, and 15 μg/ml RNase. The midi-fusion protein was purified by Ni-NTA affinity followed by gel filtration chromatography.

### Orsay VLP purification

To produce recombinant Orsay capsids containing CP-δ, ~2X10^9^ (or 2 liters) Sf21 insect cells grown in supplemented Grace’s insect medium (Life Technologies) were co-infected with 100 ml of the recombinant baculovirus expressing CP-δ and 100 ml of the recombinant baculovirus expressing CP. Cells were harvested 60 h post-infection. The cell pellets were washed with cold PBS and sonicated in a cold lysis buffer containing 50 mM Tris-HCl (pH 8.0), 300 mM NaCl, 1 mM NaN_3_, 1 mM PMSF, 10% (v/v) glycerol, 0.5% (v/v) Triton X-100, 10 μg/ml DNase, and 15 μg/ml RNase. The clarified lysate was loaded onto a Ni-NTA column. Eluted fractions were collected and further purified by a 2-ml HisTrap HP column (GE Healthcare Life Sciences).

### Electron microscopy

For EM sample preparation, FCF400-Cu grids (Electron Microscopy Sciences) were pretreated by glow-discharge at 5 mA for 1 min as previously described [56]. 5 μl of the protein solution was then added onto the grid and sat for 30 s to allow absorption. To optimize particle spread, a number of different protein concentrations ranging from 1 mg/ml to 0.01 mg/ml were prepared simultaneously. The protein solution was removed from the grids by filter paper blotting. The grids were then rinsed twice with distilled water and stained with freshly prepared 0.75% Uranyl formate solution for 60 s. After air-drying overnight, the grids were examined using a JEOL 1230 High Contrast transmission electron microscope at 80 kV. Images were recorded on a Gatan CCD detector.

### Crystallization and structure determination

Both native δ(1–66) and SeMet-substituted δ(1–66) were crystallized at 20°C by hanging-drop vapor diffusion. For native crystals, drops containing 1 μl of native δ(1–66) at 5 mg/ml concentration were combined with 1 μl of mother liquor containing 1.6 M ammonium sulfate, 0.1 M MES monohydrate (pH 6.5), and 12% (v/v) dioxane. Plate-shaped crystals appeared within 4 to 5 days. For SeMet-substituted crystals, drops containing 1 μl of SeMet-substituted δ(1–66) (5 mg/ml) were combined with 1 μl of mother liquor containing 0.1 M citric acid (pH 5.0) and 14% polyethylene glycol (PEG) 6000. Rod-shaped crystals appeared in two weeks. Crystals were cryo-protected using 25% (v/v) glycerol and flash-frozen in liquid nitrogen. Diffraction data were collected from single crystals at the Life Sciences Collaborative Access Team (LS-CAT) at the Advanced Photon Source (APS). Data were processed using HKL2000 [57].

The structure of δ(1–66) was determined by single-wavelength anomalous dispersion (SAD). The SeMet sites and experimental phases were calculated by the AutoSol Wizard in the PHENIX software suite [58]. The protein model was built with PHENIX Autobuild and COOT [59] and refined with phenix.refine. The structure was finally refined against a native dataset at 2.22-Å resolution. The final structure, which contains 284 residues and 197 waters, has a final *R*_*work*_ of 20.64% and *R*_*free*_ of 23.14%. The coordinates have been deposited in the RCSB Protein Data Bank (PDB ID: 5JIE). All structure figures were prepared using the program PyMOL unless otherwise specified (The PyMOL Molecular Graphics System, Version 1.2r3pre, Schrödinger, LLC).

### Circular dichroism

The protein sample was dialyzed into 20 mM Potassium phosphate pH 7.4, and the concentration was adjusted to 0.5 mg/mL. Circular dichroism signal was measured using a J-815 Circular Dichroism Spectropolarimeter (Jasco Analytical Instruments). The wavelength range was set from 200 nm to 280 nm with a step size of 0.2 nm. The results were analyzed using BeStSel [32].

### Mass spectrometry

A solution of 10ug/mL of protein complex in 5% ACN, 0.1% FA was directly infused at 3 uL/min with a nano-spray source into an Orbitrap Fusion Lumos (Thermo). An ionization voltage of 2200 V was used in combination with a 70V source fragmentation voltage. The intact complex was best observed with MS2 ETD 0.1 ms reaction time, Quadrupole isolation of 3300 *m/z* with a 500 *m/z* window and 15,000 resolution in an Orbitrap analyzer. Note that these conditions produce effective intact protein complex observation and do not result in substantial dissociation of the non-covalent complex or fragmentation of covalent peptide bonds. For the dissociation of the pentameric complex and observation of the intact monomer, the following conditions were used: MS2 with HCD 12% energy, 70V Source Fragmentation, 60000 Resolution Orbitrap with Quadrupole isolation of 3300 m/z with a 500 *m/z* window in high mass range, 47 scans. Complex dissociation was efficiently achieved with HCD of 9% collision energy and complete complex dissociation was observed around 12%-13% HCD collision energy. CID required much higher energies: up to 90% to see similar complex dissociation. Note the relatively high collisional energies used to achieved dissociation of the pentamer, with essentially no cleavage of covalent bonds. Orbitrap high resolution analysis (500,000 resolving power setting) allows isotopic separation of the monomer with both HCD and CID fragmentation; however, isotopic resolution of the intact 192kDa was not possible, nor expected. For isotopic resolution of the monomer the following conditions were used: MS2 with CID 50% Collision energy, 70V Source Fragmentation, 500,000 resolving power setting on the Orbitrap with Quadrupole isolation of 3300 *m/z* with a 500 *m/z* window in high mass range mode.

### Analytical ultracentrifugation

A solution of the Orsay virus full-length δ protein at 0.91 OD 280 nm (27.6 μM) was sedimented at 20°C and 30,000 rpm, and measured by UV intensity detection in a Beckman Optima XLI analytical ultracentrifuge at the Center for Analytical Ultracentrifugation of Macromolecular Assemblies at the University of Texas Health Science Center at San Antonio, using an An60Ti rotor and standard 2-channel epon centerpieces (Beckman-Coulter). All data were analyzed with UltraScan-III ver. 3.5, release 2174 (http://www.ultrascan3.uthscsa.edu). All samples were measured in a 50 mM TRIS buffer, pH 7.5, containing 250 mM NaCl. Hydrodynamic corrections for buffer density and viscosity were estimated by UltraScan to be 1.010 g/ml and 1.036 cP. The partial specific volume of the delta protein (0.7399 ml/g) was estimated by UltraScan from protein sequence analogous to methods outlined in Laue et al [60]. SV data were analyzed according to the approach described in [61]. Optimization was performed by 2-dimensional spectrum analysis (2DSA) [62] with simultaneous removal of time- and radially-invariant noise contributions [63] and meniscus fitting. After noise subtraction and meniscus fitting, the data were analyzed by the 2DSA Monte Carlo analysis to identify particle distributions in the frictional ratio–sedimentation coefficient domain [64]. The distribution suggested that a decreasing sigmoid parameterization is suitable for fitting the data with the parametrically constrained spectrum analysis (PCSA-DS), using Tikhonov regularization [65]. The calculations are computationally intensive and are carried out on high-performance computing platforms [66]. All calculations were performed on the Lonestar cluster at the Texas Advanced Computing Center at the University of Texas at Austin and on Comet and Gordon at San Diego Supercomputing Center. The resulting fit produced random residuals and is shown in S5 Fig in the Supplemental Information.

### Generation of transgenic *C*. *elegans* for Orsay reverse genetics

The plasmids pHIP_RNA1 and pHIP_RNA2 were obtained as a gift from Dr. David Wang [5]. Site mutations K43E, L44R, and ATG→CTG were introduced to the δ ORF in pHIP_RNA. 50 ng/μl mutant pHIP_RNA2, 50 ng/μl pHIP_RNA1, and 100 ng/μl pRF4 [67] were mixed and microinjected into N2 day-1 adults. Animals were cultured at 15°C on NGM plate seeded with OP50 bacteria following standard culture conditions [68]. F1 worms with the roller phenotype were picked individually to a new plate, and screened for subsequent generations of rollers to obtain stable transgenic lines. The stable transgenic line for wild-type recombinant Orsay virus was a gift from Dr. David Wang [7].

### Production of recombinant viruses

6-well RNAi plates (NGM with 1mM IPTG and 50 ng/μl Carbenicillin) seeded with *rde-1* RNAi bacteria (a clone from the Ahringer library) were prepared as described [69]. 30 L4 rollers from each stable transgenic line were picked onto a 6-well plate (5 worms/well), and cultured for 5 days at 20°C. The worms were heat induced at 33°C for 2 hours and then at 25°C for 2 days [5]. Worms were fed with IPTG-induced *rde-1* RNAi bacteria throughout the course to prevent starvation. Worms were then washed off the plates using S Basal [68]. Excess liquid was aspirated so that the volume of worm pallet and liquid was about 1:1. The mixture was then sonicated to obtain worm lysate. The crude lysate was centrifuged at 10,000xg for 10 min at 4°C. The supernatant was filtered through a 0.22 μm syringe filter and kept at 4°C till used for infection.

### Infection using recombinant viruses

*glp-4(bn2); rde-1(RNAi)* worms were used as naïve worms. The transparent intestine symptom was best observed on day-3 adults. *glp-4(bn2)* worms were used because they are sterile at high temperatures [70], and can easily grow to day-3 adults without getting starved due to progeny. *rde-1(RNAi)* were used to make the worms sensitive to Orsay infection. Synchronized L1 naïve worms were obtained by bleaching [68]. 150 L1 worms and 200 μl of viral filtrate were added to each well of a 6-well RNAi plate. The infected worms were cultured at 20°C for five days till they were day-3 adults.

### Measurement of viral infectivity

Three independent trials of infection (biological replicates) were performed. In each trial, three wells (of a 6-well plate) of worms were infected by the viral filtration from each transgenic *C*. *elegans* line. Worms from one well were counted under a stereoscope for the transparent intestine phenotype. The other two wells were used for qRT-PCR test of viral load.

For qRT-PCR, worms were washed off plates and rinsed four times with S Basal. RNA was extracted using Trizol (Invitrogen). cDNA was generated using the RETRO script Kit (Ambion). qRT-PCR was performed using PerfeCTa SYBR Green SuperMix (Quanta Biosciences), with the primers GW194, GW195 for viral RNA1 fragment and AMA-1F, AMA-1R for the internal reference gene *ama-1* [2]. The viral product was first normalized to *ama-1*, and then normalized to the values of the wild-type recombinant virus. Three technical replicates were performed for qRT-PCR, and their average was used as one data point for Fig 6D.

### Protein-competition assay

~100 synchronized L1 naïve worms were added to each well of a 96-well plate that contained 100 μl of S medium [68] with 1mM IPTG, 50 ng/μl Carbenicillin, *rde-1* RNAi bacteria, Orsay virus, and 2μg/ml Capsid, 2μg/ml δ, or 0.57 μg/ml N-terminus δ fragment δ(1–101). Theses protein concentrations were used so that the molar concentrations of the three proteins were similar. To determine the virus concentration, 2-fold serial dilution of viral filtration was conducted to determine a titration curve. The lowest viral concentration that can infect ≥70% plates was used for protein-competition. Animals were cultured on a 20°C incubator shaker till they were day-3 adults. Animals were then transferred from each well to an unseeded NGM plate to count worms with the transparency symptom. A plate with over 50% transparent worms was scored as an infected plate. The percentage of infected plates was calculated to measure viral infectivity.

## Supporting information

S1 Fig Gel filtration chromatogram of the Orsay δ proteins using a Superdex-200 column. Eluted positions for the three protein standards are indicated on top.
(TIF)

S2 Fig The predicted secondary structure of δ. Results by both PSIPRED (29) and Prof (30) are shown.
(TIF)

S3 Fig Gel filtration chromatogram of the mini-fusion protein. The protein sample was applied to a 60ml Superdex-200 column. Eluted positions for the three protein standards are indicated on top.
(TIF)

S4 Fig The full-length δ protein analyzed by mass spectrometry. (a) The homo-pentamer complex dissociates directly and exclusively to monomer upon high energy collisional activation. Only one species of 38441.12032 Da monomer was observed. This confirms that the complex is composed of five equal mass proteins that form no other complex stoichiometry. Relatively high values of HCD and CID were required to effectively fragment the complex, suggesting that the complex is very stable. The theoretical mass was calculated with the average mass of each element obtained from IUPAC. Our calculated values are similar to those obtained from available mass calculators (ExPASy, Protein Calculator v3.4). Uncertainty is calculated from IUPAC values and propagated for the intact protein. (b) High resolution mass spectrometry confirms the charge state assignment of the intact monomer mass spectrum and further supports the determined mass. The isotopically resolved spectrum results in a calculation of the monomeric mass of 38440.12 for the most abundant isotope. Note that this differs slightly from an average mass, but further confirms the previously assigned mass.
(TIF)

S5 Fig The full-length δ protein analyzed by analytical ultracentrifugation. (a) Top: Experimental data (yellow) overlayed with the genetic algorithm Monte Carlo analysis (red). Only every tenth scan is shown for clarity. Bottom: Residuals for the finite element fit shown above. (b) Sedimentation coefficient distribution obtained from the PCSA-DS analysis. A major peak centered around 5.4 s is apparent, as well as several larger species indicating possible contaminating protein species or aggregation tendency.
(TIF)

S6 Fig δ(1–66) oligomerization was disrupted by the K43E and L44R mutation. The expected position for pentamers is marked. According to the Gel filtration chromatogram, both δ(1–66)_K43E_ and δ(1–66)_L44R_ were eluted at a later position compared to δ(1–66)_WT_, suggesting the formation of monomers and possibly erroneous, smaller sized oligomers instead of pentamers. The δ(1–66)_L44R_ mutant also produced a large peak at void volume, consistent with our observation that δ(1–66)_L44R_ kept precipitating from solution during the purification process.
(TIF)

S7 Fig Multiple transgenic *C*. *elegans* lines of recombinant viruses. For each recombinant virus, over 9 independent lines of transgenic *C*. *elegans* were screened for infectivity using the transparency test. Most lines showed consistent results. Variations among lines were consistent with Jiang et al., 2014 (5). Blue indicates lines used in Fig 5.
(TIF)

S8 Fig Stereochemical constraints in the formation of potential CP-δ trimers and pentamers in the Orsay capsid. (a) An Orsay CP trimer. (b) An Orsay CP pentamer. The C-terminus of one CP molecule in the trimer and pentamer is marked with a solid back dot. The dotted curves in (a) and (b) delineate the likely paths that the downstream polypeptides need to undertake in order to form a trimeric and a pentameric CP-δ fiber, respectively.
(TIF)
