# Supplementary figures and images for: Structure of a pentameric virion-associated fiber with a potential role in Orsay virus entry to host cells
Source: PLoS Pathog. 2017 Feb 27;13(2):e1006231. doi: 10.1371/journal.ppat.1006231 (PMC5344674; doi:10.1371/journal.ppat.1006231)

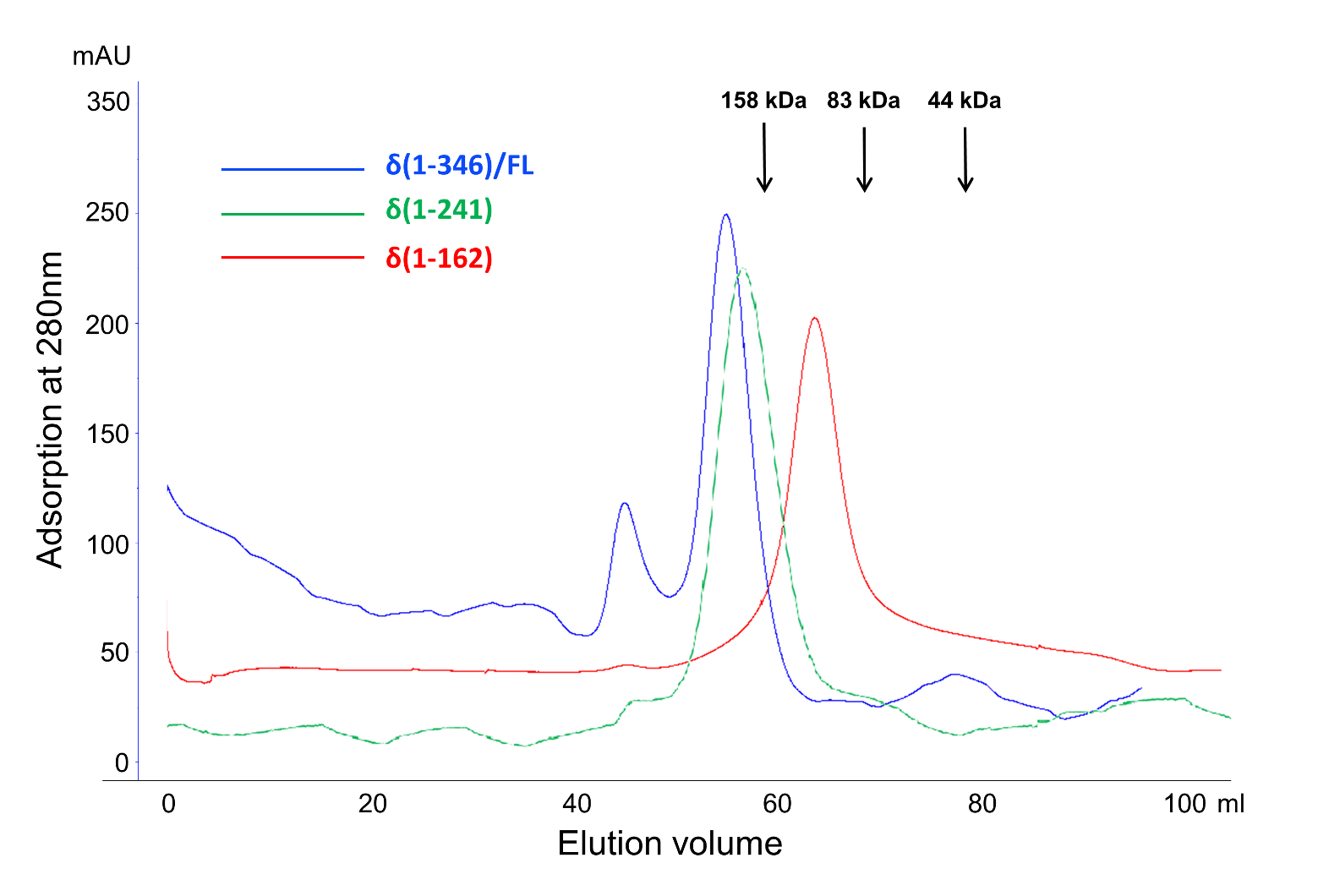

Supplement: S1 Fig — Eluted positions for the three protein standards are indicated on top. (TIF) [file ppat.1006231.s001.TIF]

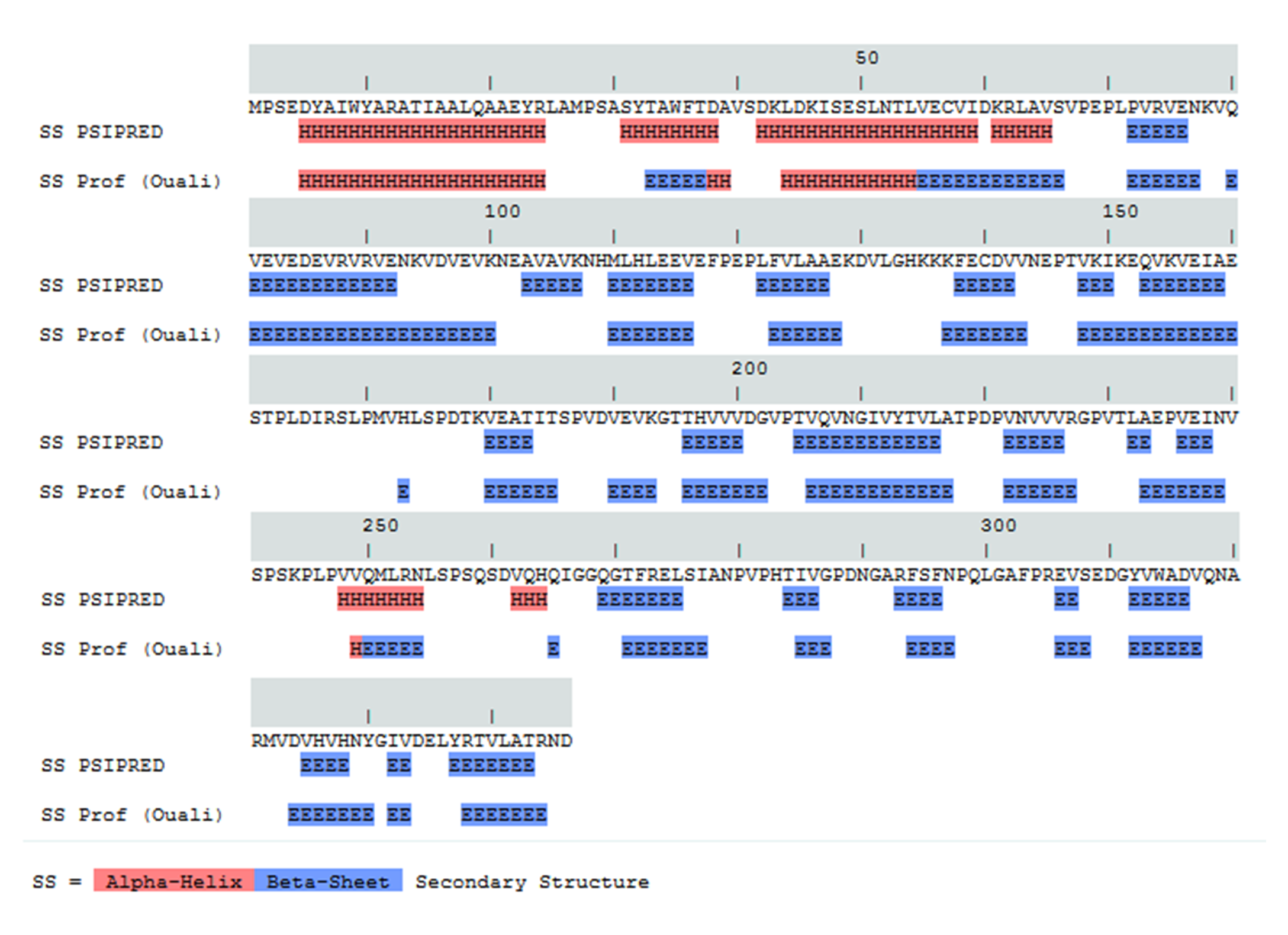

Supplement: S2 Fig — Results by both PSIPRED (29) and Prof (30) are shown. (TIF) [file ppat.1006231.s002.TIF]

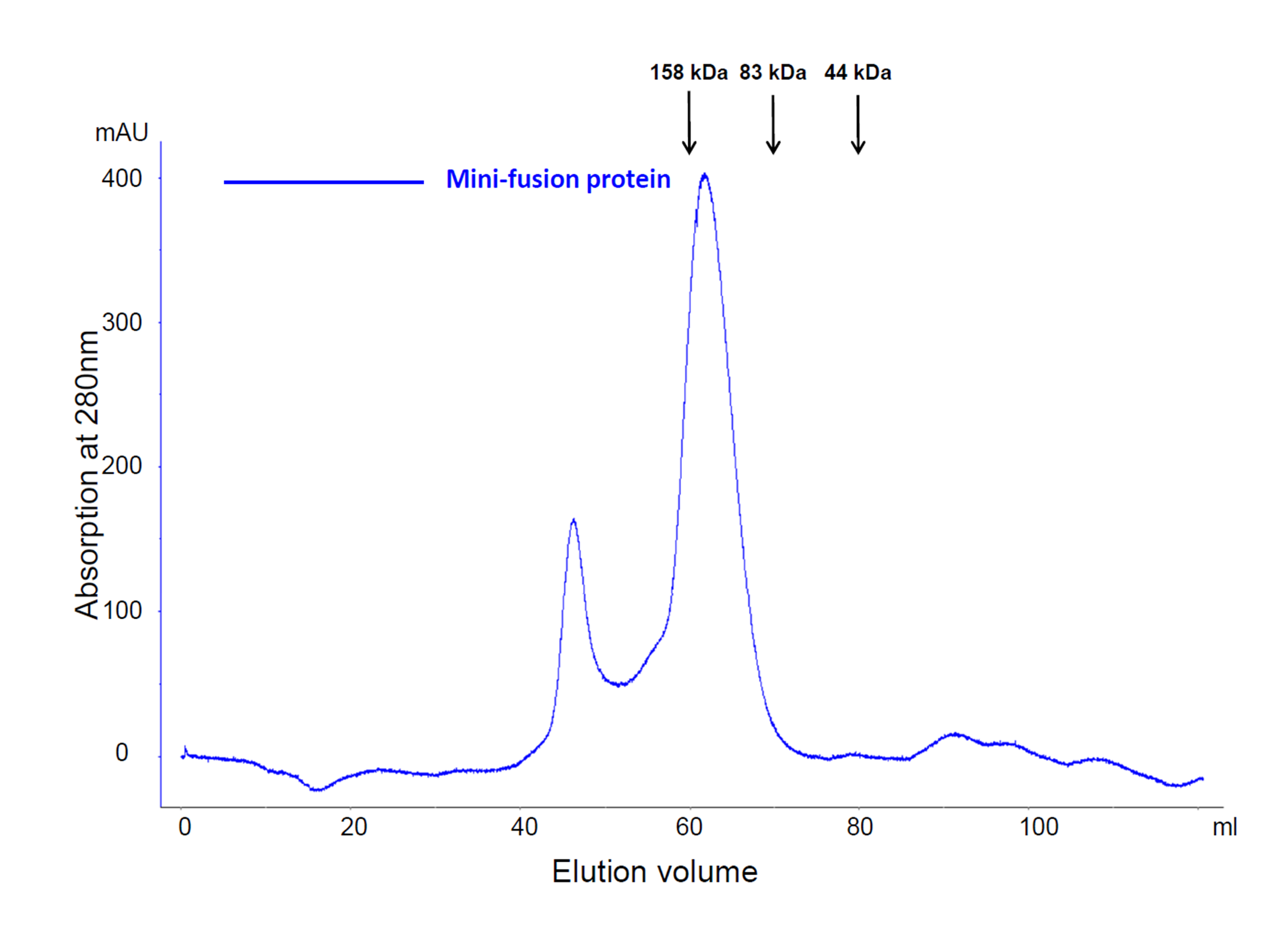

Supplement: S3 Fig — The protein sample was applied to a 60ml Superdex-200 column. Eluted positions for the three protein standards are indicated on top. (TIF) [file ppat.1006231.s003.TIF]

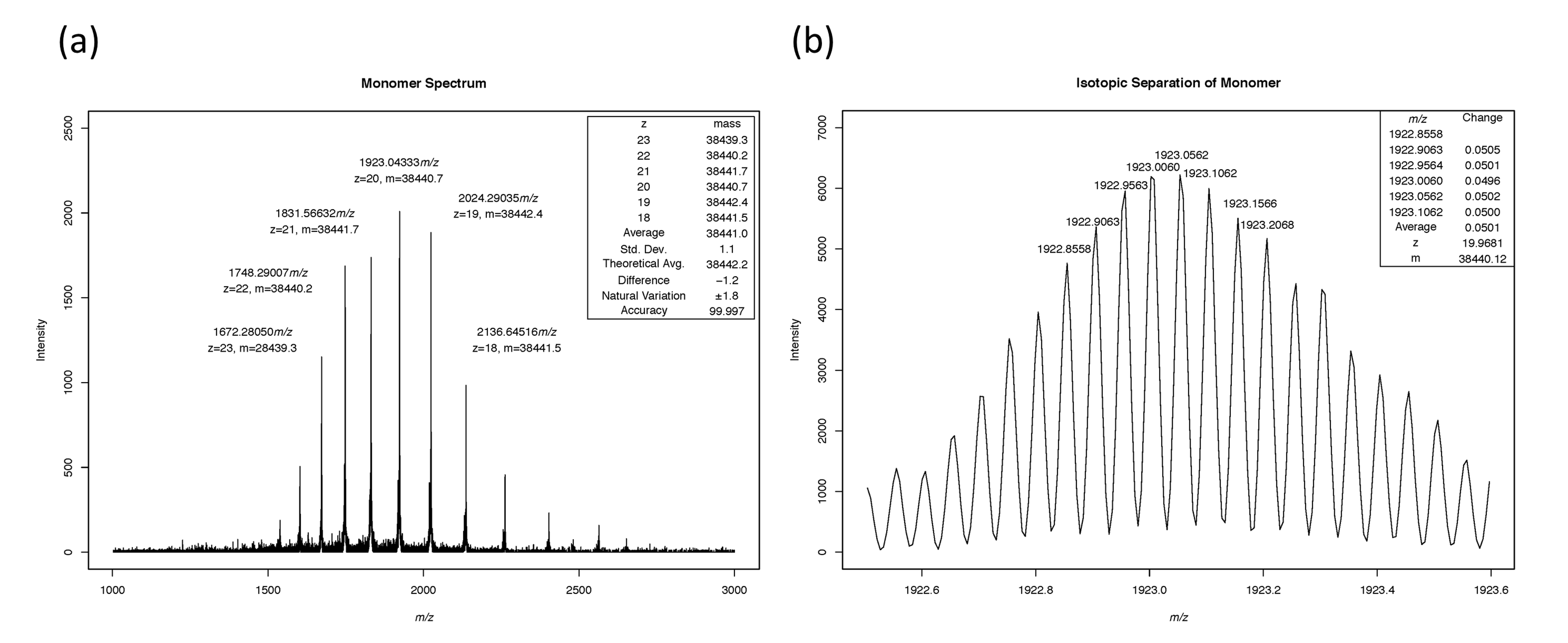

Supplement: S4 Fig — (a) The homo-pentamer complex dissociates directly and exclusively to monomer upon high energy collisional activation. Only one species of 38441.12032 Da monomer was observed. This confirms that the complex is composed of five equal mass proteins that form no other complex stoichiometry. Relatively high values of HCD and CID were required to effectively fragment the complex, suggesting that the complex is very stable. The theoretical mass was calculated with the average mass of each element obtained from IUPAC. Our calculated values are similar to those obtained from available mass calculators (ExPASy, Protein Calculator v3.4). Uncertainty is calculated from IUPAC values and propagated for the intact protein. (b) High resolution mass spectrometry confirms the charge state assignment of the intact monomer mass spectrum and further supports the determined mass. The isotopically resolved spectrum results in a calculation of the monomeric mass of 38440.12 for the most abundant isotope. Note that this differs slightly from an average mass, but further confirms the previously assigned mass. (TIF) [file ppat.1006231.s004.TIF]

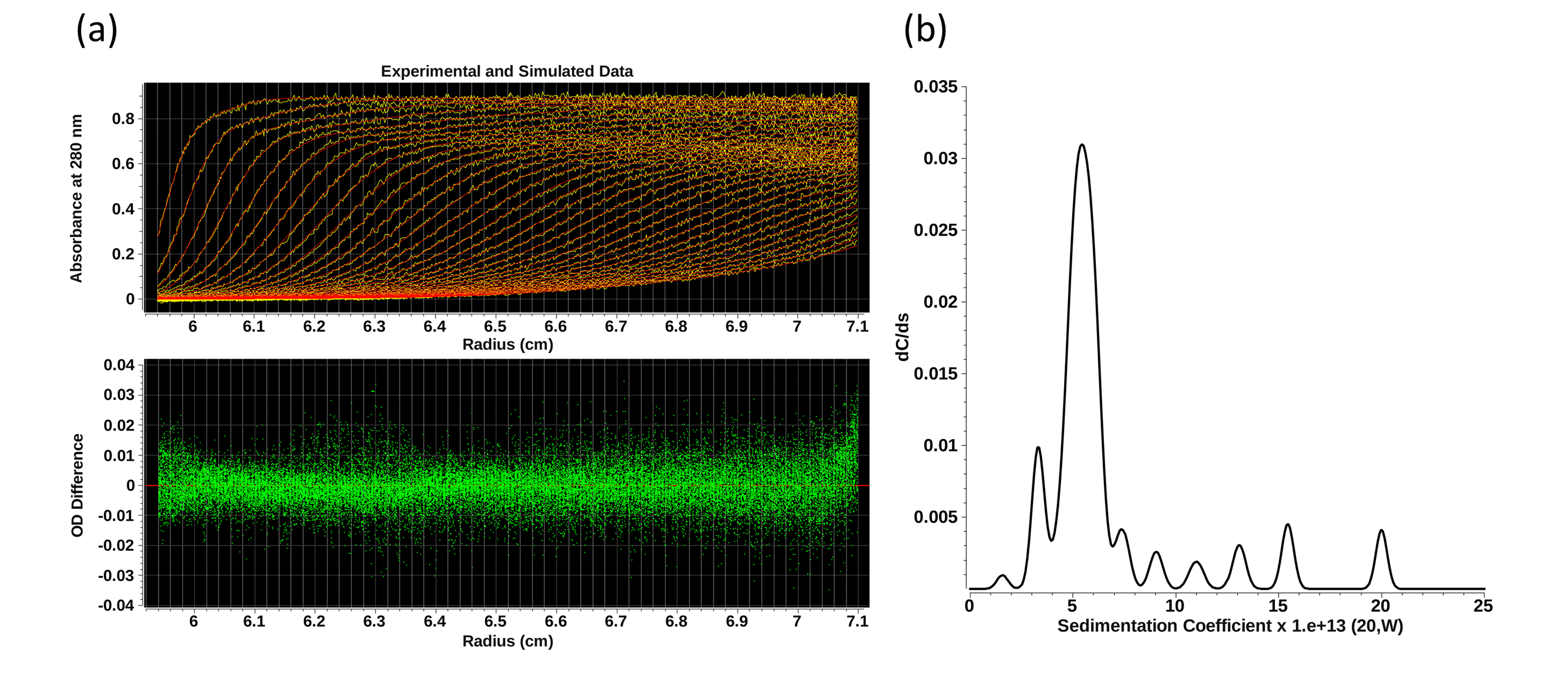

Supplement: S5 Fig — (a) Top: Experimental data (yellow) overlayed with the genetic algorithm Monte Carlo analysis (red). Only every tenth scan is shown for clarity. Bottom: Residuals for the finite element fit shown above. (b) Sedimentation coefficient distribution obtained from the PCSA-DS analysis. A major peak centered around 5.4 s is apparent, as well as several larger species indicating possible contaminating protein species or aggregation tendency. (TIF) [file ppat.1006231.s005.TIF]

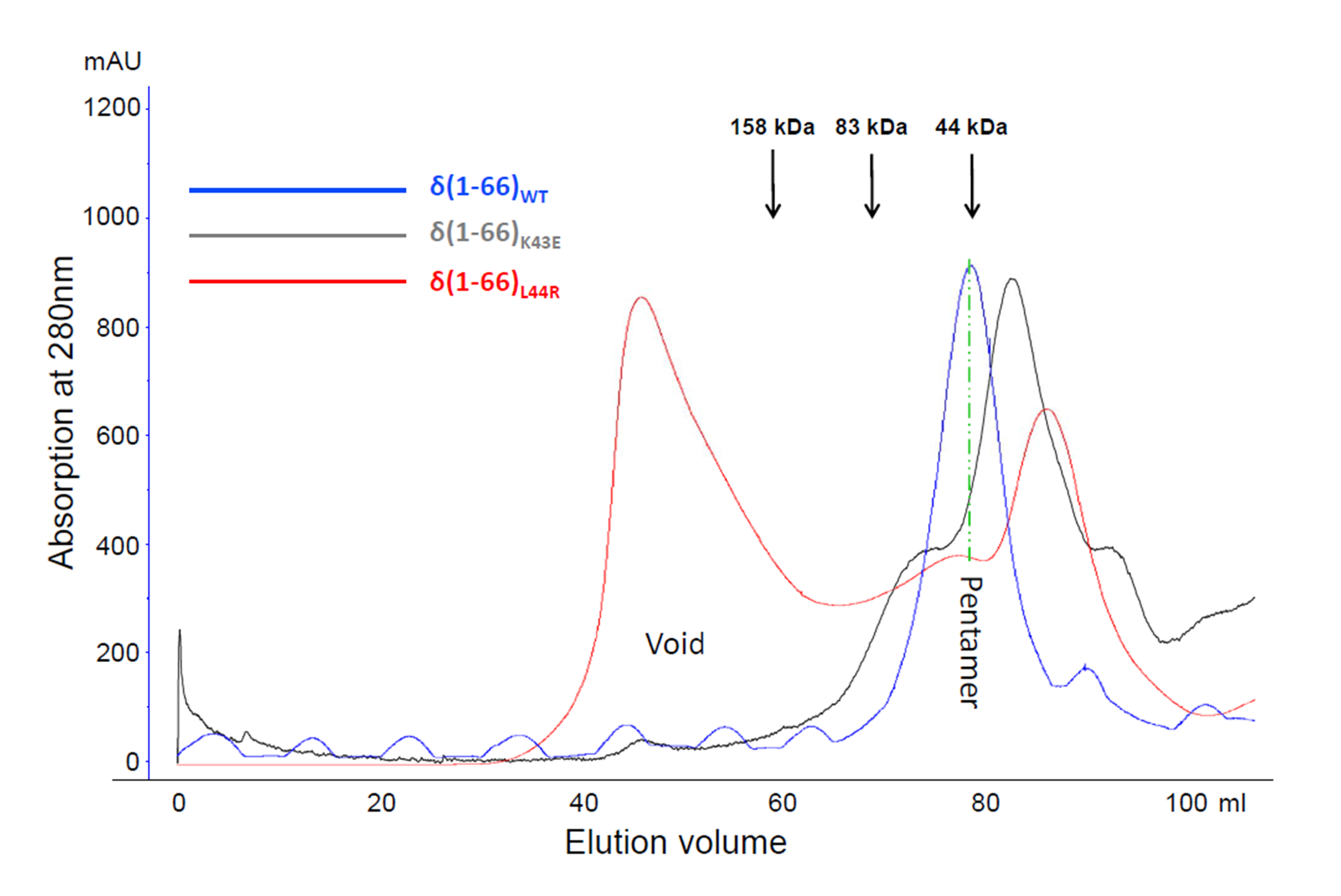

Supplement: S6 Fig — The expected position for pentamers is marked. According to the Gel filtration chromatogram, both δ(1–66)K43E and δ(1–66)L44R were eluted at a later position compared to δ(1–66)WT, suggesting the formation of monomers and possibly erroneous, smaller sized oligomers instead of pentamers. The δ(1–66)L44R mutant also produced a large peak at void volume, consistent with our observation that δ(1–66)L44R kept precipitating from solution during the purification process. (TIF) [file ppat.1006231.s006.TIF]

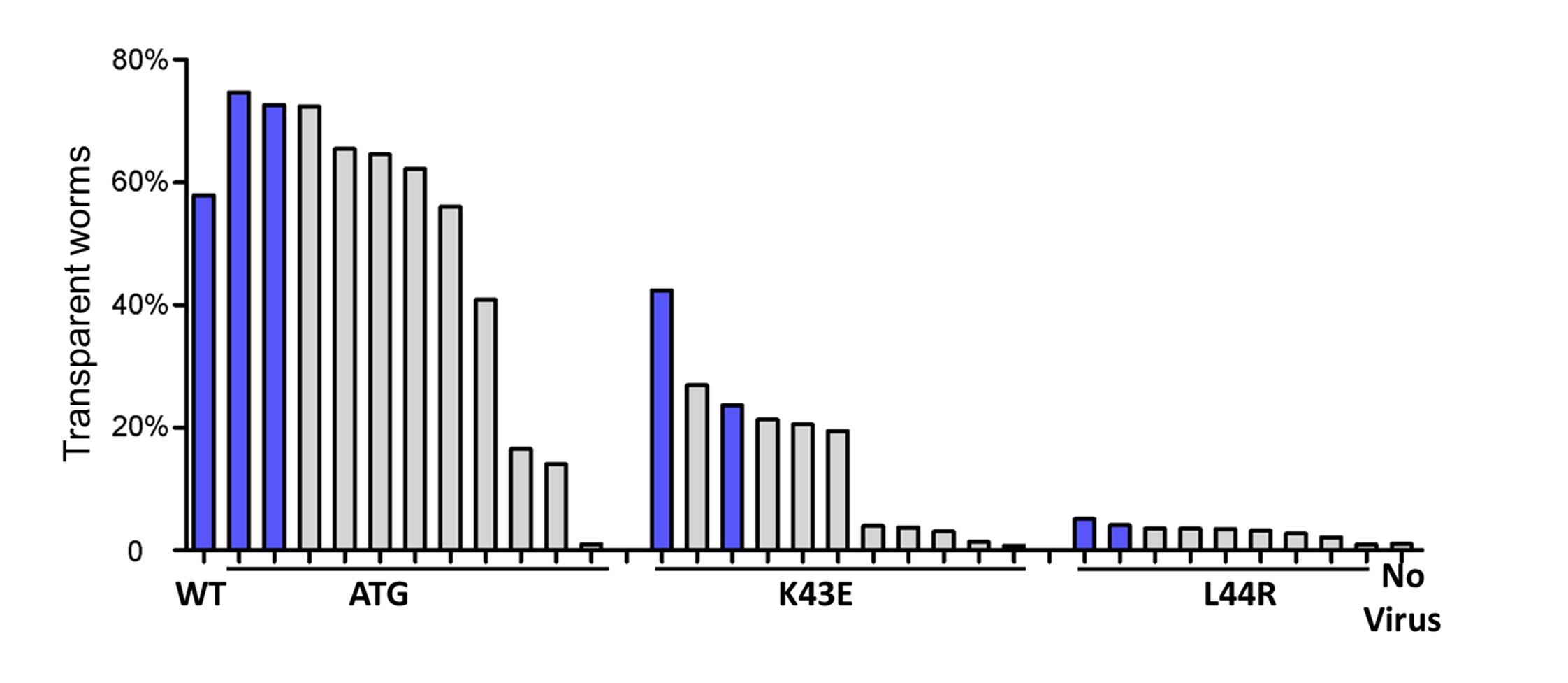

Supplement: S7 Fig — For each recombinant virus, over 9 independent lines of transgenic C. elegans were screened for infectivity using the transparency test. Most lines showed consistent results. Variations among lines were consistent with Jiang et al., 2014 (5). Blue indicates lines used in Fig 5. (TIF) [file ppat.1006231.s007.TIF]

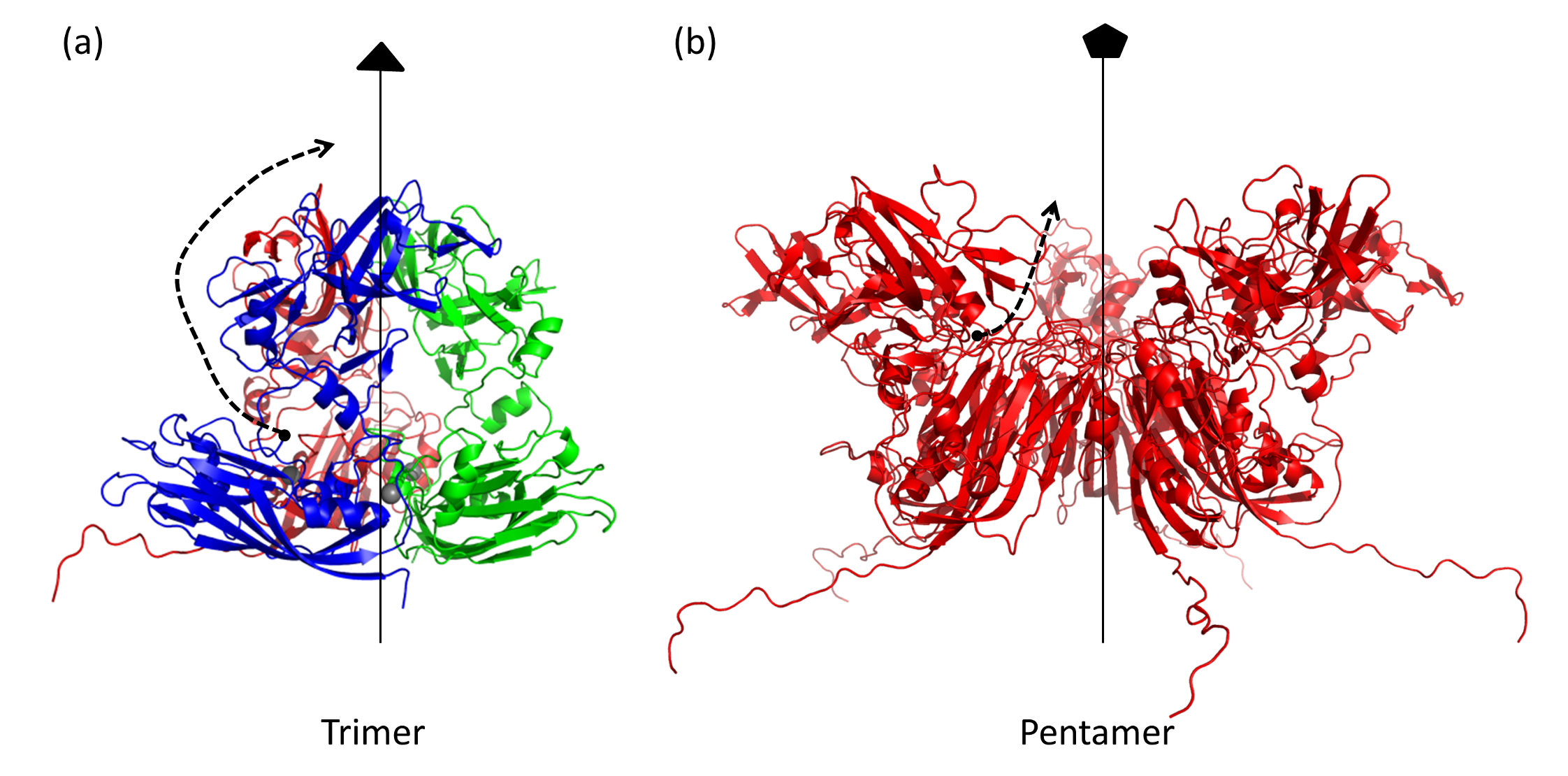

Supplement: S8 Fig — (a) An Orsay CP trimer. (b) An Orsay CP pentamer. The C-terminus of one CP molecule in the trimer and pentamer is marked with a solid back dot. The dotted curves in (a) and (b) delineate the likely paths that the downstream polypeptides need to undertake in order to form a trimeric and a pentameric CP-δ fiber, respectively. (TIF) [file ppat.1006231.s008.TIF]
